# People with Parkinson's design clinical trials charter to improve communication about, recruitment to and retention in Parkinson's clinical trials

**DOI:** 10.1186/1745-6215-16-S2-P86

**Published:** 2015-11-16

**Authors:** Jon Stamford, Tom Isaacs, Camille Carroll

**Affiliations:** 1 Cure Parkinson's Trust, London, UK; 2 Plymouth University Peninsula Schools of Medicine and Dentistry, Plymouth, Devon, UK

## Background

A 2014 survey of people with Parkinson’s (PwPs) and clinicians internationally identified the following perceived barriers to clinical trial success in Parkinson’s disease (PD): fear of potential adverse consequences; communication failures and misunderstandings over the trial process (PwPs); and insufficient financial/administrative support (clinicians)[1]. In order to address these misperceptions, we have developed a clinical trials charter for use by those considering participating in, or conducting, Parkinson’s clinical research. The ultimate aim of the charter is to improve recruitment to and retention in clinical trials in PD.

## Method

The charter has been developed internationally by PwPs, in consultation with Parkinson’s advocates, Parkinson’s clinical trials specialists and key Parkinson’s patient organisations. It is a simple two-sided document outlining standards of practice and reasonable expectations for participants and researchers. Accompanying resources, including a range of leaflets and informative film, have also been developed. The charter and resources have been reviewed and analysed in focus groups to ensure clarity of final content.

## Planned use

The charter will be evaluated in an upcoming mullticentre randomised controlled trial of simvastatin as a neuroprotective agent in PD, by means of participant surveys, to understand its potential role in supporting recruitment and retention in Parkinson’s trials.
